# Epilepsy

**Published:** 1891-05

**Authors:** 


### EPILEPSY.

(Proceedings of I. H. A. Morning Session, June 27th, 1890.)

Dr. Kimball read a paper upon epilepsy.

Dr. Carleton said:—I have never listened to a paper with more pleasure and profit than that one. It covers lots of ground and shows deep thought.

Dr. Kent—He did not repeat. He let that remedy alone and let the patient get well, and that is always a good lesson.

Dr. Kimball—Bœnninghausen said that when in epilepsy the memory was impaired he had always found the case very difficult. Has anybody here verified that?

Dr. Kent—What Bœnninghausen said, I think, was this: if the mind was impaired in the direction of true imbecility, the case was a grave one. But the memory may be impaired, and very generally is impaired in epilepsy, but that does not necessarily make a grave case. Weakness of the mind looking toward imbecility does, however, make a grave case.

Dr. Butler—I have a case of epilepsy that I would like some help on. A woman twenty-four years old, who has suffered from epilepsy since she was five years old. She came into my hands about a year ago. For a long time before that she had been under old-school treatment, and her organism had been under the influence of Bromides for years. A so-called homœopathic physician had also given her Morphine for pains here and there, and she became an habitual Morphine eater. She was taking about three grains a day and had to be given a dose before she could be persuaded to let me see her. I found the woman about twelve years old mentally. That was her last dose of Morphine, and it was a hard fight to get her out of that habit. She made things lively for the neighbors.

As her remedy I was led by a careful comparison of symptoms to select Opium, of which she received one dose and has never received another.

When she got out from under the influence of the Bromides and Morphine she commenced having convulsions every other day, and sometimes every day. She had a very peculiar aura; it was an impulse to run. She would suddenly run into another room and fall into a convulsion.

Gradually during thirteen months she has got into this condition. She has convulsions about once a week, without biting her tongue, as she formerly did. For the last three months all her symptoms are lighter but not less frequent.

General health improved most wonderfully. She has become a rather plump woman, jolly, good-natured, never cross, and she has not grown a particle mentally, but still remains as to her mind about twelve years old. She has still the same aura but lighter.

Dr. Campbell—I would suggest Belladonna.

Dr. Kimball—I think Sepia has that symptom.

Dr. Sawyer—I have treated a number of cases of epilepsy successfully, and I have never cured one without producing an eruption. An old eruption reproduced and the cure becomes simple and easy. Dr. Kimball's case is a magnificent one.

Dr. Kent—Some two years ago an epileptic patient came to me, with an eruption over the palms of the hands. The case bothered me for some time. The convulsions came both day and night, most violent in the night; they were very prolonged. I carefully selected Silicea as his remedy. They gradually became less frequent and less violent until a condition that might be called *petit mal* ensued; this improved finally into a vertigo.

At the present time he occasionally has a feeling come over him as if he would become unconscious; it amounts to an absent-mindedness. It may last two or three seconds, and then pass away. It has been ten or twelve months since the last convulsion. His mental condition is also greatly improved. He has had a continuous succession of boils ever since. He had two doses of Silicea. His age is about forty-six or forty-seven and he has had epilepsy thirty years.

Dr. Custis—I have been particularly interested in epilepsy and particularly unsuccessful in treating it. I have never cured a case, although I have thought several times that I had. One man with inherited epilepsy came to me whose case I studied very carefully. I soon found that sweet things aggravated his trouble; in fact, if I could keep him from touching anything sweet he would have no attacks. But he had an immense craving for sweet things; it was as strong as some men have for liquor. He would actually steal preserves and sweetmeats from his own people. For two years we managed to control his appetite, but the first time he ate a lot of sweet stuff in Paris he had a severe attack and died in it. One reason of my poor success is, I think, that all my patients had been saturated with the Bromide treatment. I have two patients whom epilepsy has clung to from birth up to the present time. One is sixteen the other twenty-five. Neither has ever taken any Bromides, but both Drs. Hering and Lippe failed on these cases. I have found the avoidance of sugar a help in epilepsy.

Dr. Reed—I have only had one case of epilepsy—a Swede girl. The trouble started at her fourteenth year from a fright. While playing with her sisters she touched a goose egg with a stick; it exploded with a loud noise and threw her into a fit. She would have two or three attacks in the night, and terrible ones the next day.

One dose of Calc-carb.$^{cm}$ was what I gave her. Six months of freedom from them followed, they then came back and I have never been able to control them since.

Dr. Kent—It is a perfectly proper question why we fail to cure epilepsy—sometimes because the symptoms of the disease are masked by the previous drugging, and sometimes it is difficult to find symptoms peculiar to the patient because there are so many symptoms peculiar to epilepsy which are worthless to prescribe on.

There is nothing peculiar and distinctive about epilepsy to prescribe on, but there is in every patient, if we can find them, peculiar symptoms not distinctive of epilepsy which are important as guide-posts to the simillimum. Violent screaming, sinking in the pit of the stomach, an aura in the knees, or in some particular part of the body, or an awful fear. These are peculiar and worthy of study, because they are peculiar to the patient and not peculiar to epilepsy.

On the other hand, the biting of the tongue, the fall, the frothing at the mouth, the rigidity of the muscles, are common to all cases of epilepsy and are poor things to prescribe on.

Dr. Sawyer—I do not believe there ever was an epileptic who did not have either sycosis, psora, or syphilis. Too much eating, too much work, and so forth, may be the exciting cause, but there must be a predisposing cause at bottom.

Dr. Kimball—The symptoms for Lachesis were very evident in my case. I think Dr. Kent's statement has yet to be proved. Bœnninghausen says that cases with night attacks followed by headache are almost hopeless, but we did not have at that time the proving of Bufo.

